# Women are more vulnerable to allergy than man in Gfsa city, Tunisia

**DOI:** 10.1186/1939-4551-8-S1-A256

**Published:** 2015-04-08

**Authors:** Suliman Alomar, Halim Harrath

**Affiliations:** 1 King Saud University, Saudi Arabia

## 

The prevalence of allergies has vastly increased over recent decades due to air pollution and climate change. The present study aims to identify any seasonal variations in the allergens, to help prevent and treat allergic diseases; and investigate any correlations between the reported allergens and other biotic parameters, namely age and sex. The study included 67 subjects (49 female and 18 male; age range 10-73 years) who visit the Department of Pneumology and Oto-rhino-laryngology of the Regional Hospital of Gafsa, between August 2007 and September 2008. The allergic condition of these patients was confirmed by measuring allergen specific IgE levels using the multiple allergen simultaneous test-chemoluminescent assay (MAST-CLA). A total of 30 allergens were tested and classified into seven groups: tree and grass pollen, herbaceous pollen, animal dander, moulds, latex, cockroaches and mites.

The seasonal distribution of allergens shows that the frequency of respiratory allergy was mainly observed during spring corresponding to the pollination season. However, the detection of some seasonal allergens, such as olive pollen, outside the pollination season can be explained by the cross-reactivity between allergens. The age range of patients with sensitivity was found to be between 10 and 73 years, with the most sensitive patients aged between 20 and 50 years. The sex variable was significantly associated with the studied allergens since sensitization was present more frequently in women than in men.

